# The role of microbiome-host interactions in the development of Alzheimer´s disease

**DOI:** 10.3389/fcimb.2023.1151021

**Published:** 2023-06-02

**Authors:** Christian Weber, Alexander Dilthey, Patrick Finzer

**Affiliations:** Institute of Medical Microbiology and Hospital Hygiene, University Hospital Düsseldorf, Heinrich Heine University Düsseldorf, Düsseldorf, Germany

**Keywords:** oral microbiome, oral dysbiosis, periodontal disease, Porphyromonas gingivalis, next-generation sequencing (NGS), false-positive detection, lactoferrin

## Abstract

Alzheimer`s disease (AD) is the most prevalent cause of dementia. It is often assumed that AD is caused by an aggregation of extracellular beta-amyloid and intracellular tau-protein, supported by a recent study showing reduced brain amyloid levels and reduced cognitive decline under treatment with a beta-amyloid-binding antibody. Confirmation of the importance of amyloid as a therapeutic target notwithstanding, the underlying causes of beta-amyloid aggregation in the human brain, however, remain to be elucidated. Multiple lines of evidence point towards an important role of infectious agents and/or inflammatory conditions in the etiology of AD. Various microorganisms have been detected in the cerebrospinal fluid and brains of AD-patients and have thus been hypothesized to be linked to the development of AD, including *Porphyromonas gingivalis* (PG) and *Spirochaetes*. Intriguingly, these microorganisms are also found in the oral cavity under normal physiological conditions, which is often affected by multiple pathologies like caries or tooth loss in AD patients. Oral cavity pathologies are mostly accompanied by a compositional shift in the community of oral microbiota, mainly affecting commensal microorganisms and referred to as ‘dysbiosis’. Oral dysbiosis seems to be at least partly mediated by key pathogens such as PG, and it is associated with a pro-inflammatory state that promotes the destruction of connective tissue in the mouth, possibly enabling the translocation of pathogenic microbiota from the oral cavity to the nervous system. It has therefore been hypothesized that dysbiosis of the oral microbiome may contribute to the development of AD. In this review, we discuss the infectious hypothesis of AD in the light of the oral microbiome and microbiome-host interactions, which may contribute to or even cause the development of AD. We discuss technical challenges relating to the detection of microorganisms in relevant body fluids and approaches for avoiding false-positives, and introduce the antibacterial protein lactoferrin as a potential link between the dysbiotic microbiome and the host inflammatory reaction.

## Introduction

Dementia can be described as a mostly age-associated cognitive decline, with associated deficits in the areas of memory, language, problem solving and activities of daily life. The general cause of dementia is the loss or destruction of neurons as a result of different pathological processes (Gaugler et al., 2016).

Alzheimer´s disease (AD) is a progressive neurodegenerative disease and the most common form of dementia. It slowly impairs cognition and was first described more than 100 years ago by the German psychiatrist and neurologist Alois Alzheimer (Gaugler et al., 2016).

In the US more than 10% of the population aged 65 years and over, and about one third of the population aged 85 years and over, are diagnosed with AD (Gaugler et al., 2016). In Germany 1.2 million people are diagnosed with dementia of which 50-70% fulfil the clinical criteria of AD (DGPPN, DGN, AWMF, 2016). Regarding the demographic shift and increasing life expectancy in western countries these numbers will probably grow within the next years, which represents a major challenge for health care systems (Gaugler et al., 2016).

In the context of AD, characteristic changes in the brain can be observed at the macroscopic, microscopic and molecular levels. There is a pronounced brain atrophy and, as a result, a decrease in the total weight of the brain. At the histological level, two types of protein/peptide deposits are prominently observed: Intracellular fibrils from the hyperphosphorylated, microtubule-associated protein tau (neurofibrillary tangles) and extracellular plaques from the peptides amyloid-βeta-40 and -42 (Aβ) (Fan et al., 2019; Sengoku, 2020). In addition, mitochondrial dysfunction and pronounced neuroinflammation with activation of microglia and astrocytes can be observed (Wilkins and Swerdlow, 2016).

Although most clinical trials primarily targeting Aβ and tau have not been successful (Avgerinos et al., 2021., Liu and Wang, 2019; Lu et al., 2020), a recent study indicates clinical benefit in participants with early AD under treatment with an antibody binding soluble amyloid-beta protofibrils (Lecanemab) (van Dyck et al., 2022). Even though further clinical trials are needed to determine the long-term efficacy of Lecanemab, these results provide strong support for the so-called ‘amyloid hypothesis’. However, the underlying causative mechanisms of amyloid generation and inflammation in the brain remain to be elucidated.

In this review, we discuss the potential contribution of the oral microbiome and oral infectious agents to the etiology of AD, and their link to host inflammatory conditions.

## Oral microbiota and the oral microbiome

One of several more recent hypotheses regarding the etiology of AD is a connection between AD and the oral microbiome. The oral microbiome describes the totality of microbial life in the human oral cavity. The mouth with lips, tongue, tonsils, palate, teeth, gums, supra- and subgingival space offers a variety of niches, representing a variety of ecological conditions for microorganisms. The oral microbiome is exposed to a broad set of environmental influences, including, for example, physical and chemical irritation from food intake, smoking, and dental hygiene. Next to the gastrointestinal microbiome, the oral microbiome is considered the most diverse in humans and consists of about 770 bacterial, fungal, archaeal and viral species. The oral microbiome exhibits strong inter-individual variation and also varies between different anatomical niches in the oral cavity (Fox et al., 2019; Sureda et al., 2020).

Under physiological conditions, the microbiome is thought to have a symbiotic character. Various environmental and immunological factors, however, can lead to a loss of microbial homeostasis, associated with an increase in disease-promoting, proinflammatory microorganisms and impaired immunological tolerance, which could result in tissue destruction with a systemic inflammatory response (Sudhakara et al., 2018; Bell et al., 2019; Fox et al., 2019; Sureda et al., 2020). Oral pathologies and, above all, periodontal disease (PD) as a polymicrobial, inflammatory condition involving pronounced tissue destruction could be a possible manifestation of oral dysbiosis (Bell et al., 2019; Fox et al., 2019).

## Oral pathologies are linked to the development of Alzheimer´s disease (AD)

Numerous studies have pointed towards the possibility of a connection between AD and pathologies of the oral cavity, especially PD. This connection is likely bidirectional in nature. PD refers to the inflammatory-infectious destruction of the periodontium. It is accompanied by an increase in systemic inflammatory parameters (Paraskevas et al., 2008; Passoja et al., 2010). Through systemic inflammation, PD could contribute to the development of AD (Watts et al., 2008). This could for example be mediated by a worsening of neuroinflammation (Teixeira et al., 2017), or a disruption of the blood-brain barrier (Varatharaj and Galea, 2017).

Furthermore, multiple cross-sectional studies have shown an association between tooth loss (a typical result of severe PD) and cognitive impairment, silent infarctions and changes in white matter of the central nervous system (Grabe et al., 2009; Takata et al., 2009; Minn et al., 2013; Naorungroj et al., 2013; Park et al., 2013; Ranjan et al., 2019). Other cross-sectional studies have shown an association of the clinical diagnosis of PD with cognitive decline (Martande et al., 2014; Gil-Montoya et al., 2015; Sung et al., 2019). This correlation seems more pronounced in AD-type dementias than in other dementias (Tiisanoja et al., 2019).

In accordance with cross-sectional study results, longitudinal studies have also shown an association between early tooth loss and increased risk of cognitive decline, memory impairment and dementia. Conversely, the incidence of dementia was reduced after PD treatment (Stein et al., 2007; Kaye et al., 2010; Paganini-Hill et al., 2012; Yamamoto et al., 2012; Batty et al., 2013; Reyes Ortiz et al., 2013; Okamoto et al., 2015; Stewart et al., 2015; Yoo et al., 2019). Takeuchi et al. also showed an increased risk of AD due to tooth loss, but not for vascular dementia, a form a cognitive decline caused by arteriosclerosis of brain vessels (Takeuchi et al., 2017). Similar to early tooth-loss, PD diagnosis has also been associated in longitudinal studies with an increased risk of mild cognitive impairment (MCI), dementia and AD, as well as increased cognitive decline in AD-patients (Ide et al., 2016; Chen et al., 2017; Choi et al., 2019; Iwasaki et al., 2019, Lee et al., 2017a, Lee et al., 2017b). However, these results exhibit a greater degree of inconsistency (Wu et al., 2016; Tonsekar et al., 2017), probably due to widely varying methods for determining PD (Naorungroj et al., 2013).

Regardless of the clinical diagnosis of AD, some studies have linked PD in otherwise cognitively healthy, older subjects to AD-like neuropathological changes, including increased Aβ load in the brain (Kamer et al., 2015) and increased blood amyloid and inflammatory markers (Gil-Montoya et al., 2017; Leira et al., 2020). This suggests that oral inflammatory processes may precede the development of AD.

It is assumed that PD is best understood as a consequence or expression of oral dysbiosis - defined as ‘perturbation to the structure of complex commensal communities’ leading to diseases (Petersen and Round, 2014), rather than as an infection by specific pathogens. 16S rDNA sequencing has shown that the oral microbiome in PD is fundamentally different from the oral microbiome in healthy controls, and that progression of the disease also leads to changes in microbial composition (Zhang et al, 2020). In addition, an upregulation of bacterial genes involved in proteolysis, bacterial tissue invasion and subversion of the immune system could be observed (Yost et al., 2015). It has also been demonstrated that dysbiotic conditions are associated with the increased production of virulence factors by bacteria not associated with PD under normal conditions. For example, several species of *Streptococcus*, not classified as periodontal pathogens, participate in the transcription of virulence factors in the dysbiotic community (Yost et al., 2015). Finally, the severity of oral dysbiosis is linked to the severity of PD (Zhang et al., 2020).

Oral dysbiosis towards a pro-inflammatory environment with dominance of disease-promoting species seems to emerge from the synergism between individual key players – above all the gram-negative, anaerobic bacterium *Porphyromonas gingivalis* (PG) –with other bacteria with lower individual pathogenic potential (Zhang et al., 2020). This is consistent with the fact that PG was not able to trigger PD in sterile mouse models without a pre-existing microbiome, as it apparently needs the support of other, more opportunistic microorganisms to generate dysbiosis (Darveau, 2010). These findings are supported by a recent systematic review of studies regarding changes of the oral microbiome after periodontal interventions, including the mechanical removal of plaque, antiseptics, systemic administration of antibiotics, or combinations of these methods. The authors concluded that these interventions resulted in a complex shift of the whole oral ecosystem rather than a ‘simple’ eradication of pathogens (Zhang et al., 2021).

## Infective agents involved in oral dysbiotic shift favoring AD

PG has been described as a ‘keystone pathogen’ of oral dysbiosis, meaning that it induces a shift of the symbiotic microbiota to a disease-promoting dysbiotic state, resulting in the clinical manifestation of PD (Darveau, 2010). In fact, however, the effect of PG seems to be dependent on the shift of the ecological system as a whole: The infection of germ-free mice with PG alone did not result in PD, underlining the dysbiotic nature of this disease (Darveau et al., 2012). This observation raises the question, if the colonization with PG could also be a consequence of a previous shift in the microbial community. In this scenario, an initial dysbiosis might result in optimal conditions for PG to infect the mouth. However, it was observed that infection of ‘specific-pathogen-free’ mice, which have a defined PG-free but otherwise normal oral microbiome, with PG resulted in a dysbiotic shift of the microbiome and later PD-associated alveolar bone loss (Darveau et al., 2012), consistent with the theory that PG can contribute to the development of PD.

In this sense, PG has also been described as a ‘pathobiont’. This term refers to a microorganism that typically exists in a synergistic, non-pathogenic state in its host, but which has the potential of becoming pathogenic under certain conditions. In the case of PG it has been postulated that a low-abundance colonization of the mouth with PG may not be pathogenic. The accumulation of bacterial plaque, however, for example because of poor oral hygiene, may lead to the initiation of innate immune systems defense mechanisms, resulting in a first state of inflammation. At a certain point, this inflammation ‘awakens’ PG in its pathogenic potential, which then starts to promote inflammation and a microbial shift, for example by infecting mucosal dendritic cells resulting in an uncontrolled inflammation mediated by the adaptive immune system (Meghil and Cutler, 2020). PG thus likely exerts its effects dependent on opportunistic microbes. Furthermore, the effect of PG also likely depends on the host immune response, with PG alone unable to induce PD-associated alveolar bone loss in mice deficient of specific CD4+-cells or several cytokines (Baker et al., 1999).

In addition to its connection to oral dysbiosis, PG is also directly associated with AD. In a retrospective analysis, 2355 participants over 60 years from the third National Health and Nutrition Examination Survey (NHANES-III) were screened for an association between cognitive performance tests and serum levels of PG-IgG-antibodies. Comparing individuals with the highest and lowest levels of PG-IgG, people with higher levels were more likely to have impaired delayed verbal recall and serial subtraction capabilities. This association of poor cognitive performance and PG-antibodies remained robust after adjusting for socioeconomic and vascular variables (Noble et al., 2009). However, in a case-cohort study from the same working-group with 219 subjects, higher serum levels of PG-IgG were not associated with incident AD. Nevertheless, elevated levels of antibodies against the periodontal, dysbiosis-associated bacteria *Actinomyces naeslundii* and *Eubacterium nodatum* were associated with an elevated risk for AD (Noble et al., 2014).

Later on, post mortem brain samples of AD-patients and neurologically normal individuals were immunohistochemically studied for the PG-protease gingipain, showing a significant correlation of the abundance of gingipain with AD diagnosis and the abundance of tau protein. This is especially interesting, as the same group also showed, that tau can be a target of gingipain proteolysis (Dominy et al., 2019). The proteolysis of tau is thought to generate toxic fragments, involved in AD pathology (Boyarko and Hook, 2021).

Additionally, Dominy et al. identified PG-DNA in AD brains and in the brains of gingipain-positive brain samples from patients without diagnosed dementia by using quantitative PCR. In a similar way, PG-DNA was also detected in the cerebrospinal fluid and saliva of ten non-deceased subjects with mild to moderate cognitive impairment, diagnosed with probable AD. They detected PG-DNA in seven of ten cerebrospinal fluid samples and ten of ten saliva samples (Dominy et al., 2019).

In experimental studies mice exposed intraperitoneally to the lipopolysaccharide (LPS) of PG developed learning and memory impairment, Aβ accumulation, and microglia-mediated neuroinflammation (Wu et al., 2017). In addition, oral administration of PG to mice triggered Aβ production, neurodegeneration and neuroinflammation (Ilievski et al., 2018). Vice versa, the administration of a small-molecule inhibitor of gingipain in mice orally infected with PG led to reduced amyloid production and neuroinflammation and was associated with increased survival of hippocampal neurons (Dominy et al., 2019).

The detection of PG-DNA and proteins in the central nervous system may be accounted for the presence of PG in the central nervous system. This however, raises the question, how the bacterium can be translocated to the brain? Bacterial outer-membrane vesicles are an alternative explanation for the presence of bacterial components in the central nervous systems. They can contain microbial genetic material, LPS and virulence factors. In the case of PG, outer-membrane vesicles have been shown to also contain gingipain (Castillo et al., 2022). A potential role of the protease gingipain or other virulence factors in the pathogenesis of AD is thus conceivable even in the absence of viable PG in the central nervous system (Nara et al., 2021). Nevertheless, false-positive detections are also a possible explanation for the presence of PG-DNA in the brain.

In addition to PG, other classes of bacteria that have the potential to generate bacterial amyloids and biofilms have been identified, especially comprising the phylum *Spirochaetota* and its member genus *Treponema*. *Spirochaetes*, including *Treponema*, are known to form biofilms through the synthesis of amyloids, enabling the formation of aggregated colonies in the central nervous system. This phenomenon is classically associated with neuro-lues and neuro-borreliosis by *Treponema pallidum* and *Borrelia burgdorferi* (Miklossy, 2016). What is more, *Spirochaetes* seem to be able to produce Aβ protein precursor (AβPP) and AβPP-like proteins as well (Miklossy, 1993). When formed *in-vitro*, spirochetal biofilms contain these main components of senile AD plaques. Vice versa, spirochetal DNA was found in senile plaques (Miklossy, 2016). The oral cavity is a rich source of different *Treponema* lineages, many of which are associated with the pathogenesis of PD (Zeng et al., 2021). *Treponema* DNA could be found post-mortem in the cortex of AD patients, as well as in the trigeminal ganglia, potentially implying a scenario of oral *Treponema* spp. being transported *via* cranial nerves (Riviere et al., 2002).

It is important to note that extracellular DNA is a structural component of bacterial biofilms (Whitchurch et al., 2002). Both spirochetal biofilms and senile plaques contain AD-associated proteins as well as spirochetal DNA. The potential of a direct connection between senile plaques and bacterial biofilms has therefore been proposed (Miklossy, 2016).

## Oral dysbiotic signatures are associated with the development of cognitive decline and AD

There are several studies investigating a possible connection between the oral microbiome community structure and AD. Through a MEDLINE search (search terms: ‘oral microbiota’, ‘oral microbiome’ and ‘alzheimer’, ‘dementia’, ‘cognitive’, ‘cognition’), we identified 13 studies investigating the connections between AD and the oral microbiome (Cockburn et al., 2012; Liu et al., 2019; Bathini et al., 2020; Holmer et al., 2021; Wu et al., 2021; Yang et al., 2021; Chen et al., 2022a; Chen et al., 2022b; Chen et al., 2022c; Cirstea et al., 2022; Fu et al., 2022; Taati Moghadam et al., 2022; Tadjoedin et al., 2022). For Chen et al. 2022a and Cirstea et al., 2022, full-text manuscripts could not be obtained. For these studies, the following synopsis is based on information available in the abstracts. The details of all studies are summarized in **Table 1**.

**Table 1:** Studies investigating the oral microbiome of people with AD, dementia, or cognitive impairment.

Author	Design	Participants	Control	Dementia diagnostic	Sampling	Sequencing	Additional measurements	Main results
Cockburn et al., 2012	Cross-sectional	n=10 (5 dementia, 3 cognitive impaired non-dementia, 2 cognitively healthy)	Cognitive impaired non-dementia and cognitively healthy people	Neurocognitive testing, Geriatric depression scale	Subgingival	16s, Illumina, V3	none	- No significant difference in alpha-diversity (number of OTUs) - Higher abundance in dementia-group of Bacteroidetes (phylum), Prevotellaceae (family), Prevotella (genus) - Lower abundance in dementia-group of Fusobacteria (phylum), Fusobacteriaceae (family), Fusobacterium, Leptotrichia (genus) - No significant differences between different periodontal pockets of the same individual but with varying pocket depth
Liu et al., 2019	Cross-sectional	n=78 (39 AD, 39 cognitively healthy)	Cognitively healthy people	?	Saliva	16s (Illumina ?)	APOE4-genotyping	- Lower alpha-diversity in AD-group (method?) - Higher abndance in AD-group of Moraxella, Leptotrichia, Sphearochaeta (genus) - Lower abundance in AD-group of Rothia (genus) - Significant association of APOE4-genotype with higher abundance of Abiotrophia and Desulfomocrobium and lower abundance of Actinomyces and Actinobacillus (genus)
Bathini et al., 2020	Cross-sectional	n=81 (17 AD, 21 mild cognitve impairment, 43 cognitively healthy)	MCI and cognitively healthy people	MMSE, CDR	Saliva	16s, Illumina, V3-4	APOE4-genotyping, salivary inflammmatory marker, olfactory capacity	- No significant difference in alpha-diversity (method?) - Higher abundance in MCI-group of Leptotrichia wadei, Cardiobacterium valvarum (species) - Lower abundance in AD-group of Filifactor villosus, Filifactor alocis, Porphyromonas gingivalis, Prevotella tannerae (species) - No significant difference in inflammatory markers
Holmer et al., 2021	Cross-sectional	n=106 (35 AD, 36 MCI, 35 subjectice cognitive decline)	MCI people and people with subjective cognitive decline	MMSE, Winblad-Criteria for MCI/SCD	Subgingival, supragingival	16s, Illumina, V3-4	Periodontal pocket depth, bleeding on probing, X-ray examination of alveolar bone loss	- Higher alpha-diversity in AD-group (Shannon-Index) - Higher richness in AD-group (number of OTUs) - Higher abundance in AD-group of Lachnospiraceae (family), Prevotella oulorum, Slackia exigua (species) - Lower abundance in AD-group of Actinomyces (genus), Rothia aeria, Corynebacterium durum (species)
Wu et al., 2021	Cross-sectional	n=230 (52 AD, 51 MCI, 51 subjective cognitive decline, 76 cognitively healthy)	MCI people, people with subjective cognitive decline and cognitively healthy people	MMSE	Supragingival	PacBio, full-length-analysis V1-9	Decayed, missing, and filled teeth index	- Lower alpha-diversity in AD-group (Shannon-/Simpson-Index) - Lower richness in AD-group (number of OTUs) - Higher abundance in AD-group of Firmicutes (phylum), Lactobacillales, Actinomycetales, Veillonellales (order), Lactobacillaceae, Streptococcaceae, Actinomycetaceae, Veillonellaceae (family), Lactobacillus, Streptococcus, Actinomyces, Veillonella (genus) - Lower abundance in AD-group of Bacteroidetes, Fusobacteria (phylum), Fusobacteriales, Cardiobacteriales (order), Porphyromonadaceae, Cardiobacteriaceae (family), Porphyromonas, Fusobacterium, Cardiobacterium (genus)
Yang et al., 2021	Cross-sectional	n=35 (17 AD, 18 cognitively healthy)	Cognitively healthy people	MMSE, CDR, neuropsychiatric inventory questionnaire	Saliva	16s, Illumina, V3-4	Perceived stress, salivary inflammatory marker, cathepsin B and cortisol, serum CRP, leukocytes and albumin, geriatric oral health assessment indes, decayed, missing, and filled teeth index, gingival-bleeding, periodontal pocket depth, Loss of attachement, plaque index	- Lower alpha-diversity in AD-group (Shannon-Index) - Lower richness in AD-group (number of OTUs, Chao 1) - Higher abundance in AD- and MCI-group of Firmicutes, Actinobacteria (phylum) - Lower abundance in AD-group of Patescibateria, Synergistetes (phylum), Porphyromonas, Prevotella (genus) - Higher level of salivaty inflammatory markers in AD-group
Chen et al., 2022a	Longitudinal	n=66 (66 AD)	none (comparison before and after a 24-week oral health intervention and routine care)	MMSE, neuropsychiatric Inventory, nursing home adjustment scale, AD cooperative study-ADL	Subgingival	16s (Illumina ?)	Kayser-Jones brief oral health status examination	?
Chen et al., 2022b	Cross-sectional	n=60 (60 AD)	none (division into three groups of 20 by the dietary inflammatory index (from most-antiinflammatory diet (PT1 (oral)/FT1 (fecal)) to most pro-inflammatory diet (PT3/FT3)	MMSE	Subgingival, supragingival, fecal	16s, Illumina, V3-4	Food frequency questionnaire, international physical activity questionnaire, serum inflammatory markers	- No significant difference in alpha-diversity and richness (number of OTUs, Shannon-/Simpson-Index, Chao 1, ACE) - Higher abundance in PT1 compared to PT3 of Prevotella, Olsenella (genus) - Higher abundance in PT3 compared to PT2 of Abiotrophia, Neisseria, Parvimonas (genus) - Higher abundance in FT1 compared to FT3 of Alistipes, Ruminococcus, Odoribacter, unclassified Firmicutes (genus) - Higher abundance in FT1 compared to FT2 of Pseudoxanthomonas, Firmicutes, Bacillariophyta, Oxalobacter, Alistipes, Rhodocyclaceae (genus) - No significant difference in inflammatory markers between all three groups - Higher IL4-level in patients with severe
Chen et al., 2022c	Cross-sectional	n=172 (43 mild AD, 89 moderate AD, 40 cognitively healthy)	Cognitively healthy people	MMSE, CDR, CMRT/CCT, bloodmarkers, APOE-/PS1-genotyping*, Amyloid-PET* (*in a selective manner, if routine diagnostic was unabale to identify AD)	Subgingival, fecal	16s, Illumina, V3-4	none	- Lower fecal alpha-diversity in AD-group, no significant difference in oral alpha-diversity (Simpson-/Shannon-Index) - Higher fecal richness and lower oral richness in AD-group (numberof OTUs, Chao 1, ACE) - Higher diversity and lower richness or oral microbiota compaered to fecal - Higher oral abundance in AD-group of Firmicutes, Fusobacteria (phylum), Selenomonadales (order), Veillonellaceae, Streptococaceae, Leptotrichiaceae (family), Selenomonas, Veillonella, Streptococcus, Leptotrichia (genus) - Lower oral abundance in AD-group of Proteobacteria (phylum), Gammaproteobacteria (class), Aggretibacter, Lautropia (genus) - Higher fecal abundance in AD-group of Proteobacteria, Verrucomicrobia, Actinobacteria (phylum), Gammaproteobacteria, Verrucomicrobiae, Actinobacteria (class), Enterobacteriales, Bifidobacteriales, (order), Enterobacteriaceae, Actinomycetaceae (family), Escherichia_Sigella, Allermansia, Lactpbacillus, Streptococcus, Bifidobacterium (genus) - Lower fecal abundance in AD-group of Firmicutes, Bacteroidetes (phylum), Erysipelotrichia (class), Erysipelotrichales (order), Acidaminococcaceae, Bacteroidaceae (family), Ruminococcus2, Phascolarctobacterium, Clostridium_IV, Bacteroides, Parabacteroides (genus) - Higher oral abundance in moderate AD compared to mild AD of Firmicutes (phylum), Erysipelotrichia (class), Erysipelotrichiales, Coriobacteriales (order), Lactobacillaceae, Erysipelotrichiaceae, Coriobacteriaceae (family), Anaeroglobus, Lactobacillus, Stomatobaculum, Schwartzia, Atopobium, Solobacterium (genus) - Lower oral abundance in moderate AD compared to mild AD of Proteobacteria (phylum), Pseudomonadales (order), Pseudomonadaceae (familiy), Aggregatibacter, Pseudomonas, unclassified_Pasteurellaceae (genus) - Higher fecal abundance in moderate to mild AD of Synergistetes, Proteobacteria, Actinobacteria (phylum), Synergistia, Gammaproteobacteria (class), Synergistales, Pasteurellales, Enterobacteriales, Actinomycetales (order), Synergistales, Pasteurellaceae, Enterobacteriaceae, Micrococcaceae (family), Stenotrophomonas, Proteus, Escherichia_Shigella, Rothia, Alloprevotella (genus) - Lower fecal abundance in moderate AD compared to mild AD of Firmicutes (phylum), Sutterellaceae (family), Ezakiella, Olsenella (genus) - Oral Firmicutes (phylum) and fecal Proteus (genus) showed negative correlation with MMSE - Oral Proteobacteria showed positive correlation with MMSE
Cirstea et al., 2022	Cross-sectional	n=108 (54 AD, 54 cognitively healthy)	Cognitively healthy people	?	Oral (?), fecal	16s (Illumina ?)	?	- Higher oral and lower fecal alpha-diversity in AD-group (method ?) - Higher oral abundance in AD-group of Weeksellaceae (family), Porphyromonas gingivalis (species) - Lower oral abundance in AD-group of Streptococcaceae, Actinomycetaceae (family)
Fu et al., 2022	Cross-sectional	n=40 (20 AD, 20 cognitively healthy)	Cognitively healthy people	MMSE, CDR	Saliva	16s, Illumina, V3-4	Probing depth, clinical attachment level, gingival recession, plaque index (PI), and residual teeth, serum Aβ42, pTau, Tau, inflammatory marker, anti-PG-LPS-antibody	- No significant difference in alpha-diversity and richness (Shannon-Index, number of OTUs) - Higher abundance in AD-group of Capnocytophaga sp ora clone DZ074, Eubacterium infirmum, Prevotella buccae, Selenomonas artemidis (species) - Lower abundance in AD-group of Streptococcus mutans, Rothia dentocariosa (species)
Taati Moghadam et al., 2022	Cross-sectional	n=30 (15 AD, 15 cognitively healthy)	Cognitively healthy people	MMST, CMRT, bloodmarkers	Mucosa(buccal?), subgingival, supragingival, lingual, teeth surface	Quantitative realt-time PCR for periodontal pathogens	Serum inflammatory markers	- No diversity-quantification - Higher abundance in AD-group of Porphyromonas gingivalis, Fusobacterium nucleatum, Prevotella intermedia (species) - Higher level of inflammatory marker in AD-group
Tadjoedin et al., 2022	Cross-sectional	n=28 (14 cognitive impaired, 14 cognitively healthy)	Cognitively healthy people	MMSE, Hopkins Verbal Learning Test	Subgingival	16s, Illumina, V3-4	Clinical attachment loss, periodontal pockets, gingival bleeding	- No significant difference in alpha-diversity and richness (Shannon-Index, number of OTUs) - Higher abundance in cognitive impaired group of Porphyromonas, Treponema (genus), Porphyromonas gingivalis, Treponema denticola (species)
		mean=80						

The included studies were published between 2012 (Cockburn et al., 2012) and 2022 (Chen et al., 2022a). The number of participants ranged from ten (Cockburn et al., 2012) to 172 (Chen et al., 2022c) with a mean of 72 participants.

Twelve studies had a cross-sectional design with one examination where a group of patients (cases) with AD, dementia, or cognitive impairment is compared to a control group of healthy individuals. Five studies (Cockburn et al., 2012; Bathini et al., 2020; Holmer et al., 2021; Yang et al., 2021; Chen et al., 2022c) additionally included a third group of MCI patients, or people with subjective cognitive decline, or further differentiated into mild and moderate AD (Chen et al., 2022c). Chen et al. (b) had no healthy control group, but divided their AD patients into three groups regarding the patients’ nutritional habits (Chen et al., 2022b), based on the so-called ‘dietary inflammatory index’ (DII) (Phillips et al., 2019). One study employed a longitudinal design without a control group, but with a comparison of the oral microbiota before and after a 24-week oral health intervention (Chen et al., 2022a). Therefore, the studies of Chen et al., 2022a; Chen et al., 2022b) are excluded from the discussion regarding possible differences between patients and healthy controls. Nevertheless, they should be mentioned in the broader context of the oral microbiome in AD.

For the diagnosis of dementia and the exclusion of dementia/cognitive impairment in the healthy controls, eleven studies used the mini mental status examination (MMSE) as a clinical diagnostic in accordance with current guidelines (DGPPN, DGN, AWMF, 2016). Two studies additionally used cranial MRT (magnetic resonance tomography) or CT (computer tomography) (Chen et al., 2022c; Taati Moghadam et al., 2022).

The material used, to investigate the oral microbiome varied across the studies. Four studies used saliva (Liu et al., 2019; Bathini et al., 2020; Yang et al., 2021; Fu et al., 2022), seven used subgingival samples (Chen et al., 2022a; Chen et al., 2022b; Chen et al., 2022c), Cockburn et al., 2012; Holmer et al., 2021; Taati Moghadam et al., 2022; Tadjoedin et al., 2022), four used supragingival samples (Holmer et al., 2021; Wu et al., 2021; Chen et al., 2022b; Taati Moghadam et al., 2022), and three studies included additional fecal samples (Chen et al., 2022b; Chen et al., 2022c; Cirstea et al., 2022). Moghadam et al. also included samples from the buccal mucosa, the tongue and the teeth surface (Taati Moghadam et al., 2022).

For interrogating the structure of the oral microbiome, all included studies, with the exception of Taati Moghadam et al., 2022, relied on 16S rDNA sequencing. Briefly, 16S rDNA sequencing involves amplifying and sequencing the ubiquitous 16S ribosomal RNA gene (16S rDNA), followed by identification of the detected sequences in databases. 16S rDNA sequencing enables a characterization of the biological diversity and the individual abundances of oral bacteria (Suárez Moya, 2017). Except for Wu et al., the included studies employed the Illumina short-read sequencing platform, typically targeting the V3–V4 hypervariable regions of the bacterial 16S rRNA gene (Suárez Moya, 2017). Wu et al. carried out a full-length analysis (V1-9) of the bacterial 16S rRNA gene using the PacBio sequencing platform (Wu et al., 2021). Moghadam et al. performed a quantitative real-time PCR for periodontal pathogens (Taati Moghadam et al., 2022), limited to quantifying the abundances of pre-specified bacterial species.

Four studies found no difference in the alpha diversity between cases and controls (Cockburn et al., 2012; Bathini et al., 2020; Fu et al., 2022; Tadjoedin et al., 2022). Chen et al., 2022b also found no difference in alpha diversity between AD patients with different dietary inflammatory indices (DIIs) (Chen et al., 2022b). By contrast, four studies found a lower alpha diversity in cases compared to controls (Liu et al., 2019; Wu et al., 2021; Yang et al., 2021; Chen et al., 2022c), while two studies found a higher alpha diversity (Holmer et al., 2021, Cirstea et al., 2022). The results of the included studies with respect to alpha diversity were thus not consistent.

With respect to beta diversity, all studies identified differentially abundant taxonomic groups between case and control groups. It is important to note, however, that comparability of the studies was limited by varying sampling locations in the mouth (saliva, sub-/supragingival, teeth, tongue, buccal mucosa) and differences in control groups (cognitively healthy, people with subjective cognitive decline, mild cognitive impaired). Also, the statistical methods to define significant differences between groups varied, with three studies (Liu et al., 2019; Chen et al., 2022c; Fu et al., 2022) including linear discriminant analysis of effect size (LEfSe) (Segata et al., 2011) and only four studies (Cockburn et al., 2012; Yang et al., 2021; Chen et al., 2022c; Tadjoedin et al., 2022) explicitly mentioning the use of a multiple testing correction method, important for compositional analysis of the microbiome (Gloor and Reid, 2016).

The included studies’ results with respect to differentially abundant taxonomic groups are presented in detail in **Table 1**. It is important to note a general lack of consistency across studies. This may be due to the limited size or heterogeneity of the included study populations; some reported results may also be due to contamination (also see the next section on reducing the rate of false-positive detections in sequencing-based studies). In addition, species considered as oral pathogens were identified at both higher (e.g. *Slackia exigua, Porphyromonas gingivalis, Capnocytophaga* sp.*, Eubacterium infirmum, Prevotella buccae, Fusobacterium nucleatum, Prevotella intermedia, Treponema denticola*, all associated with oral diseases (Spratt et al., 1999; Maestre et al., 2007; Hiranmayi et al., 2017; Mohanty et al., 2019)) and lower (*Filifactor villosus, Filifactor alocis, Prevotella tannerae, Streptococcus mutans*, *Porphyromonas gingivalis* (Hajishengallis and Lamont, 2012; Hajishengallis et al., 2012; Lemos et al., 2019; Mohanty et al., 2019; Jiang et al., 2021; Könönen et al., 2022)) abundances in cases compared to controls across studies. What is more, sometimes the same species (e.g. *Porphyromonas gingivalis*) was detected at both higher and lower abundances in different studies.

Having noted these limitations, we briefly summarize the included studies’ results in the following paragraphs.

At the phylum level, taxa found at higher abundance in cases include *Firmicutes* (Wu et al., 2021; Yang et al., 2021; Chen et al., 2022c), *Actinobacteria* (Yang et al., 2021), and *Fusobacteria* (Chen et al., 2022c). By contrast, some studies found a lower abundance of *Fusobacteria* (Wu et al., 2021), as well as of *Patescibacteria, Synergistetes* (Yang et al., 2021) and *Proteobacteria* (Chen et al., 2022c) in cases.

At the genus level, a higher abundance in cases was found for *Prevotella* (Cockburn et al., 2012), *Moraxella* (Liu et al., 2019)*, Leptotrichia* (Liu et al., 2019; Chen et al., 2022c), *Sphearochaeta* (Liu et al., 2019), *Lactobacillus, Actinomyces* (Wu et al., 2021), *Streptococcus, Veillonella* (Wu et al., 2021; Chen et al., 2022c), *Selenomonas* (Chen et al., 2022c), *Porphyromonas, Treponema* (Tadjoedin et al., 2022), *Kingella, Mogibacterium* and *Gemella* (Liu et al., 2019). Lower abundances were found for *Fusobacterium* (Cockburn et al., 2012; Wu et al., 2021), *Rothia* (Liu et al., 2019), *Actinomyces* (Holmer et al., 2021), *Porphyromonas* (Wu et al., 2021; Yang et al., 2021), *Cardiobacterium* (Wu et al., 2021), *Prevotella* (Yang et al., 2021), *Aggretibacter, Pseudomonas* and *Lautropia* (Chen et al., 2022c).

Finally, at the species level, cases showed higher abundances of *Leptotrichia wadei, Cardiobacterium valvarum* (Bathini et al., 2020), *Prevotella oulorum, Slackia exigua* (Holmer et al., 2021), *Porphyromonas gingivalis* (Cirstea et al., 2022, Taati Moghadam et al., 2022; Tadjoedin et al., 2022), *Capnocytophaga* sp. *oral clone DZ074, Eubacterium infirmum, Prevotella buccae, Selenomonas artemidis* (Fu et al., 2022), *Fusobacterium nucleatum, Prevotella intermedia* (Taati Moghadam et al., 2022) and *Treponema denticola* (Tadjoedin et al., 2022). By contrast, lower abundances could be found for *Filifactor villosus, Filifactor alocis, Porphyromonas gingivalis, Prevotella tannerae* (Bathini et al., 2020), *Rothia aeria, Corynebacterium durum* (Holmer et al., 2021), *Streptococcus mutans* and *Rothia dentocariosa* (Fu et al., 2022).

In summary, no clear oral microbiome signature of AD could so far be identified. Results with respect to PG, characterized as a keystone pathogen of oral microbiome dysbiosis (see above), were also not consistent. Furthermore, an important limitation of the summarized studies is the potentially confounding role of reverse causality. For example, shifts in the oral microbiome structure of AD patients may be partly explained by changed patterns of personal oral hygiene due to the onset of dementia or pre-dementia (Naorungroj et al., 2013).

Nevertheless, the fact that all included studies found significant differences between cases and controls suggests a potentially important role of oral microbiome dysbiosis in the pathogenesis of AD. Pathogenicity of oral pathogens is not necessarily linked to high abundance (Meghil and Cutler, 2020), and microbial phenotypes, linked to e.g. virulence factors not detectable by 16S rDNA sequencing, may also play an important role.

## Reducing the false-positive detection rate in sequencing-based studies

Both the existence of a ‘brain microbiome’ – that is, the microbial colonization of the brain under normal physiological conditions – as well as the colonization of the brains of neurodegenerative disease patients by specific pathogens such as PG have remained controversial (Link, 2021). Interrogating samples with low or no bacterial biomass with respect to the presence of specific bacterial species or bacterial community structure is well-known to be a challenging problem (also evident in the controversies around the existence of placental and blood microbiomes (Castillo et al., 2019; de Goffau et al., 2019; Blaser et al., 2021) potentially affected by multiple methodological biases that may lead to false-positive detections (Eisenhofer et al., 2019). This includes the so-called ‘contaminome’ – the contamination of DNA extraction and sequencing kits with bacterial DNA fragments (Salter et al., 2014; Glassing et al., 2016; Chrisman et al., 2022), as well as bioinformatic challenges, e.g. with respect to the reliable assignment of 16S sequencing data (Edgar, 2018) and the contamination of reference databases (Lu and Salzberg, 2018; Steinegger and Salzberg, 2020). Due to the additional amplification step, studies based on the 16S rDNA sequencing are generally more prone to false-positives than studies based on metagenomic shotgun sequencing. On the other hand, the presence of large quantities of host genetic material may reduce the sensitivity of metagenomic approaches. Recommendations (Eisenhofer et al., 2019) to reduce false-positive detection rates include (1) the use of negative controls, (2) characterization of the ‘contaminomes’ of the utilized reagents, (3) validation of potential detections using different DNA extraction and sequencing kit, (4) combination of different microbiome sequencing approaches, such as 16S rDNA sequencing and metagenomic sequencing and (5) the application of contamination detection algorithms (Liu et al., 2022).

In the case of 16S rDNA sequencing, full-length 16S rDNA sequencing approaches (Curry et al., 2022) may exhibit reduced false-positive rates. In the case of shotgun metagenomics, assessing the k-mer spectrum (Breitwieser et al., 2018) or the uniformity of horizontal reference genome coverage of the reads assigned to a taxon can be used to effectively filter out false-positive detections associated with nonspecific assignments, driven e.g. by eukaryotic repeat motifs, and reference database contamination.

## Lactoferrin and its influence on oral health, PG and the central nervous system

In addition to the presence, absence and differential abundance of different microbes, host factors may also play an important role in the emergence of oral dysbiosis. Lactoferrin (LF) is a multifunctional human protein present on several mucosal surfaces and in several body fluids as milk and tears that may represent a possible link between the oral microbiome, PG and the central nervous system. It exhibits structural similarities to transferrin and is capable of binding iron (Fe3+), the iron-saturated form of LF being referred to as ‘holo-LF’ and the iron-free form referred to as ‘apo-LF’. LF fulfils a broad spectrum of different functions in the body (Wang et al., 2019). For example, it improves intestinal iron-absorption by binding Fe3+, followed by binding to a specific LF-receptor and internalization (Lönnerdal, 1994; Lönnerdal et al., 2011). It also functions as an antioxidant (Faridvand et al., 2017), reduces bacterial growth (Lynge Pedersen and Belstrøm, 2019; Lu et al., 2021), inhibits the invasion of viruses (Miotto et al., 2021) and modulates inflammatory processes (Puddu et al., 2011).

Salivary LF plays a key role in maintaining the health and general homeostasis of the mouth. It is produced in the salivary glands and its production is upregulated during states of infection and inflammation (Glimvall et al., 2012). LF is capable of inhibiting typical oral pathogens including bacteria (e.g. *Streptococcus mutans, Porphyromonas gingivalis, Aggregatibacter actinomycetemcomitans*), viruses (e.g. *Herpes simplex*) and fungi (e.g. *Candida albicans*) (Lynge Pedersen and Belstrøm, 2019).

Especially with respect to PD, LF fulfils key functions inhibiting disease initiation and progression. For example, biofilms on the tooth surfaces support the growth of proinflammatory bacteria, leading to lesions of the mucosal connective tissue and gingival bleeding. As a consequence, the availability of hemoglobin and iron in the oral fluids is increased, contributing to the growth for key periodontal pathogens, such as PG, and further reinforcing the inflammatory process. By binding free iron, LF inhibits the growth of bacterial pathogens, leading to the downregulation of the inflammatory loop and reduction of oral pathology (Lynge Pedersen and Belstrøm, 2019).

Interestingly, LF also seems to play a role in maintaining iron homeostasis in the central nervous system. AD patients show increased concentrations of iron in their brain (Smith et al., 2010; Yang et al., 2022). Disturbed iron metabolism may result in neuronal dysfunction and neuronal death, as iron and other metals are associated with potential neural toxicity and trigger increased lipid-peroxidation (Liu et al., 2018).

Similar to the oral upregulation of LF under inflammatory conditions, LF can also be upregulated in the central nervous system, mediated by LF secretion by microglia. Probably in response to increased free iron concentrations in the brains of AD patients, the LF concentration in the brains of these patients is also elevated (Liu et al., 2018). A transcriptomic analysis of neuropathological samples found that upregulated transcription of LF best differentiated between AD patients and controls and showed the highest correlation to Aβ-pathology. Also, an involvement of LF in the processing of APP could be demonstrated. Together, this suggests, that increased transcription of LF in the central nervous system is a core feature of AD pathology (Tsatsanis et al., 2021).

Lactoferrin’s antibacterial activity may be of particular relevance in the context of its activity against PG. Several studies were able to identify mechanisms by which LF inhibits PG: It (1) is able to bind PG and other oral pathogens (Kalfas et al., 1991), (2) can agglutinate PG and inhibits its auto aggregation relevant for its biofilm formation (Soukka et al., 1993), (3) inhibits the growth of PG by binding and withdrawing iron as a growth factor (Aguilera et al., 1998), (4) removes the hemoglobin receptor on the cell surface of PG, disrupting the iron metabolism (Shi et al., 2000), (5) inhibits the adhesion of PG and other oral pathogens on surfaces (Arslan et al., 2009; Wakabayashi et al., 2009) and (6) inhibits the proteinases of PG, functioning as key pathogenic factors in PD (Dashper et al., 2012).

On the other hand, PG and other oral pathogens are able to bind and degrade LF (Alugupalli and Kalfas, 1996; de Lillo et al., 1996). In addition, PG can induce the expression of micro-RNA-584 in human gingival epithelial cells, which can inhibit the expression of LF-receptors and reduces the anti-inflammatory effect of LF (Ouhara et al., 2014). The existence of these direct anti-LF mechanisms is consistent with the theory of LF as a central protective factor against PG. Interestingly, it has been shown, that AD-patients have reduced salivary concentrations of LF (Carro et al., 2017), which might be a consequence of the degradation of LF by PG or other dysbiotic mechanisms, resulting in a vicious cycle of insufficient defense and an increasingly inflammatory environment (Olsen and Singhrao, 2020).

Regarding the occurrence of PG and gingipain in the central nervous system of AD patients (Dominy et al., 2019), it was recently hypothesized that the increased availability of free iron in AD brains (Smith et al., 2010; Yang et al., 2022) may attract the migration of PG, as PG requires iron for its growth (Olsen, 2021). In this scenario, increased concentrations of LF in the AD brain could be interpreted as a protective mechanism against free iron and, potentially, PG migration.

## Salivary lactoferrin as a non-invasive diagnostic tool for Alzheimer`s disease

The discovery of reduced concentrations of LF in the saliva of AD patients prompted several studies investigating the potential use of salivary LF in the diagnosis of AD. Compared to established diagnostic liquor parameters and imaging methods (DGPPN, DGN, AWMF, 2016), which are highly invasive and/or cost-intensive, the collection of saliva for diagnostic purposes can be implemented in a non-invasive and cost-effective manner.

Reduced levels of LF were first observed in the saliva of patients with oral dryness (xerostomia) (Mizuhashi et al., 2015). Xerostomia is a typical feature of the aging population, associated with a poorer oral health status and malnutrition. The condition can be seen as a sign of an altered function of the salivary gland (Xu et al., 2019).

Reduced salivary LF was found to better discriminate between cases and controls than tau and beta-amyloid-42 in the liquor in patients with AD and mild cognitive impairment (Carro et al., 2017). Reduced levels of salivary LF were also found to correlate with PET-measured amyloid load and to enable a differentiation between AD and frontotemporal dementia with a sensitivity of 87% and a specifity of 91% (González-Sánchez et al., 2020). In addition, Reseco et al. reported an association between reduced levels of LF and higher PET-measured amyloid load in the brain of even cognitively healthy, elderly people (Reseco et al., 2021).

These promising results notwithstanding, however, replication of the diagnostic utility of LF in additional studies and by independent research groups is required. A first study trying to reproduce the diagnostic use of salivary LF failed to find a correlation between salivary LF, clinical status and liquor parameters (Gleerup et al., 2021).

## Therapeutic applications of lactoferrin

Several clinical studies have investigated whether the described antimicrobial and oral homeostasis-promoting properties of LF enable its use – typically in the form of bovine LF, which is found in cow’s milk and which exhibits a high degree of similarity to human LF – as a therapeutic agent against oral pathologies and oral microbiome dysbiosis (Rosa et al., 2021).

At least four studies (Daly et al., 2019, Ishikado et al., 2010; Nakano et al., 2016; Nakano et al., 2019) investigated the influence of LF in tablets, or toothpaste on clinical signs of PD, showing a positive impact on several clinical indices, inflammatory conditions and oral malodor. Six studies (Kondo et al., 2008; Shimizu et al., 2011; Shin et al., 2011; Adams et al., 2017; Morita et al., 2017; Nakano et al., 2017) also included microbiological parameters. In these papers, LF additionally to again positively influencing clinical parameters, lowered the number of PG, *Prevotella intermedia* and *Fusobacterium nucleatum* and promoted a presumed beneficial shift of the oral microbiome. The details of these studies are presented in **Tables 2**, **3**.

**Table 2:** Studies on the therapeutic effects of LF on clinical signs of PD.

Author	Participants	Intervention	Control	Duration	Measurements	Main results
Ishikado et al., 2010	n=12 (subjects with multiple sites of more than 3mm probing depth (as PD sign))	Tablet with 180mg liposomal, bovine LF Once per day	None	4 weeks	Probing depth, bleeding on probing, gingival crevicular fluid volume, levels of TNFα, IL-1β, IL-6, monocyte chemoattractant protein-1 (MCP-1) in gingival crevicular fluid, PG-LPS induced production of TNFα, IL-6, IL-1β, MCP-1 and Toll-like-receptor 2 and 4 mRNA expression in isolated peripheral blood mononuclear cells	- Reduction in probing depth compared to baseline (p = 0.0226) - Reduction in MCP-1 compared to baseline(p <0.001) - Reduction in PG-LPS induced production of TNFα (<0.05), IL-6 (p <0.05), IL-1β (p <0.01) and MCP-1 (p <0.01) compared to baseline
Nakano et al., 2016	n=39 (subjects with oral malodor (as PD sign))	Tablet with 20mg LF, 2.6mg lactoperoxidase, 2.6mg glucose oxidase Once per day	Placebo	Immediate measurement after 10min and 30min in a crossover design	Volatile sulfur compounds, H2S, CH3SH in breath	- Lower concentration of volatile sulfur compounds in breath in LF group compared to placebo after 10min (p = 0.002) - Lower concentration of H2S in breath in LF group compared to placebo after 10min (p <0.001)
Daly et al., 2019	n=229 (healthy subjects)	Toothpaste containing LF, lactoperoxidase, glucose oxidase Usage twice daily	Commercial toothpaste	12 weeks, after initial dental prophylaxis and 4 week use of the commercial toothpaste	Plaque index, modified gingival index, bleeding index	- Decrease in modified gingival index in LF group (p <0.001) and commercial toothpaste group (p <0.001) compared to baseline - Decrease in bleeding index (p = 0.021) and plaque index (p <0.001) in LF group compared to baseline - Superior improvement of plaque index (p <0.001), modified gingival index (p <0.001) and bleeding index (p <0.001) in LF group compared to commercial toothpaste group
Nakano et al., 2019	n=109 (healthy subjects)	Tablet with 20mg LF and 2.6mg lactoperoxidase, or 60mg LF and 7.6mg lactoperoxidase Once per day	Placebo	12 weeks	Plaque index, gingival index, oral health impact profile	- Decrease in gingival index in high-dose LF group compared to baseline (p <0.001) - Superior reduction of gingival index in high-dose LF group compared to placebo (p = 0.027) - Decrease in plaque index in high-dose (p = 0.02) and low-dose (p = 0.024) LF group compared to baseline

**Table 3:** Studies on the therapeutic effects of LF on microbiological parameters.

Author	Participants	Intervention	Control	Duration	Measurements	Results
Kondo et al., 2008	n=18 (subjects with mild chronic PD)	Tablet with LF (dosage unclear) Three times per day	Placebo	12 weeks	Clinical periodontal conditions, number of total bacteria, PG and Prevotella intermedia in the subgingival plaque and saliva, level of human and bovine LF in gingival crevicular fluid and saliva, level of endotoxin in gingival crevicular fluid and saliva	- Superior reduction of number of total bacteria, PG and Prevotella intermedia in subgingival plaque in LF group compared to placebo (p ?) - Higher level of bovine LF in gingival crevicular fluid and saliva in LF group compared to placebo (p ?)
Shimizu et al., 2011	n=72 (subjects with chronic PD)	Tablet with 100mg LF, 1.8mg lactoperoxidase, 24.3mg glucose oxidase Three times per day	Placebo	12 weeks	Plaque index, gingival index, probing depth, clinical attachment loss, plaque control record, bleeding on probing, number of PG in saliva and subgingival plaque, level of human and bovine LF in gingival crevicular fluid and saliva, level of endotoxin in gingival crevicular fluid and saliva	- Higher level of bovine LF in gingival crevicular fluid and saliva in LF group compared to placebo (p <0.05) - No significant difference in changes of clinical and microbiological parameters between groups
Shin et al., 2011	n=15 (subjects with oral malodor (as PD sign))	Tablet with 100mg LF, 1.8mg lactoperoxidase, 24mg glucose oxidase Two tablets with 1h between	Placebo	Immediate measurement after 10min, 1h and 2h in a crossover design	Volatile sulfur compounds, H2S, CH3SH in breath, number of total bacteria, lactobacilli, total streptococci, mutans streptococci, Aggregatibacter actinomycetemcomitans, PG, Prevotella intermedia, Fusobacterium nucleatum, terminal restriction fragment length polymorphism analysis on oral bacteria	- Lower concentration of volatile sulfur compounds in breath in LF group compared to placebo after 10min (p = 0.049) - Lower concentration of CH3SH in breath in LF group compared to placebo after 10min (p = 0.011) - Lower copy number at 2h for terminal restriction fragment length polymorphism analysis 265 bases (belonging to Prevotella, Porphyromonas, Streptococcus, Treponema, Eubacterium, Clostridiales, Bacteroidales, Desulfomicrobium) in LF group compared to placebo (p = 0.033)
Morita et al., 2017	n=46 (31 nursing home residents, 15 healthy older individuals with tongue coating)	Tablet with 20mg LF, 2.6mg lactoperoxidase, 2.6mg glucose oxidase One tablet after every meal	Placebo	8 weeks	Plaque control record, probing depth, bleeding on probing, tongue coating score, halitosis and dry mouth (as oral mucosal wettability of buccal mucosa and tongue), volatile sulfur compounds in breath, number of total bacteria and periodontal pathogenic bacteria	- Decrease in total number of bacteria (p <0.01) and PG (p <0.05) in supragingival plaque in LF group compared to baseline - Decrease in total number of bacteria (p <0.01), PG (p <0.05) and Fusobacterium nucleatum (p <0.05) in tongue coating in LF group compared to baseline - Decrease in total number of bacteria (p <0.01) and increase in number of Fusobacterium nucleatum (p <0.05) in placebo group compared to baseline - Superior reduction of number of PG in LF group compared to placebo (p <0.05) - Decrease in probing depth (p <0.05), bleeding on probing (p <0.05), tongue coating score (p <0.01) and oral mucosal wettability (p <0.05) in both groups compared to baseline
Adams et al., 2017	n=111 (healthy subjects)	Toothpaste containing LF, lactoperoxidase, glucose oxidase, amyloglucosidase, lysozym, IgG Usage twice daily	Commercial toothpaste	14 weeks after 4 week use of the commercial toothpaste	16S rDNA sequencing of supragingival plaque	- Shift in the microbial community in LF-group compared to baseline (p = 0.01) - Difference in the microbial community between LF group and commercial toothpaste after 14 weeks (p = 0.011) - Increase in abundance of 12 bacterial species associated with oral health (p <0.001) and decrease in abundance of 11 bacterial species associated with PD (p <0.01) in LF-group - Largest increase inLF group for Neisseria flava (p <0.001), largest decrease in Rothia dentocariosa (p <0.001) - Increase in abundance of one bacterial species associated with oral health (p <0.001) and decrease in abundance of four bacterial species associated with PD (p <0.01) in commercial toothpaste group
Nakano et al., 2017	n=47 (healthy older individuals with tongue coating)	Tablet with 20mg LF, 2.6mg lactoperoxidase, 2.6mg glucose oxidase One tablet after every meal	Placebo	8 weeks	Plaque control record, probing depth, bleeding on probing, volatile sulfur compounds in breath, 16S rDNA sequencing of supragingival plaque and tongue coating	Supragingival plaque: - Lower alpha diversity (Shannon-Index, Chao 1, PD whole tree) of supragingival plaque in LF group compared to placebo after 8 weeks (p <0.05) - Increase in abundance in supragingival plaque in LF group of human intestinal firmicute CS13 (p <0.05), Streptococcus sp. oral taxon 071 (p <0.05), Bifidobacterium pseudocatenulatum (p <0.05), Bifidobacterium longum (p <0.05), Ruminococcus gnavus (p <0.05), Roseburia inulinivorans (p <0.05), Clostridium leptum (p <0.05), Streptococcus sp. oral taxon 057 (p <0.05), bacterium IARFR575 (p <0.05), bacterium ic1379 (p <0.05) (species) - Decrease in abundance in supragingival plaque in LF group of Clostridium aminophilum (p <0.05), Campylobacter gracilis (p <0.05), Prevotella oris (p <0.05), Capnocytophaga gingivalis (p <0.05), Treponema sp. oral taxon 231 (p <0.05), Leptotrichia sp. oral taxon 212 (p <0.05), Enterobacter sp. AR451 (p <0.05), Prevotella buccae (p <0.05), Neisseria cinerea (p <0.05), Prevotellaceae bacterium DJF_LS10 (p <0.05) (species) - Increase in abundance in supragingival plaque in placebo group of Terrahaemophilus sp. oral taxon G25 (p <0.05), Haemophilus sp. T13 (p <0.05) (species) - Decrease in abundance in supragingival plaque in placebo group of Streptococcus salivarius (p <0.05) (species) Tongue coating: - No significant difference in alpha diversity of tongue coating between groups (Shannon-Index, Chao 1, PD whole tree) - Increase in abundance in tongue coating in LF group of Streptococcus mitis (p <0.05), Gemella haemolysans (p <0.05), Megasphaera micronuciformis (p <0.05), Actinomycetaceae bacterium 'ARUP UnID 87' (p <0.05), Streptococcus tigurinus (p <0.05), Bifidobacterium longum (p <0.05), Faecalibacterium prausnitzii (p <0.05), Ruminococcus sp. CB3 (p <0.05), Lactobacillus gasseri (p <0.05), Lactobacillus salivarius (p <0.05), Gemella sp. oral taxon G07 (p <0.05), Eubacterium rectale (p <0.05), Veillonella parvula (p <0.05), Neisseria sp. ChDC B321 (p <0.05), Bacteroides sp. S327 (p <0.05) (species) - Decrease in abundance in tongue coating in LF group of Lachnospiraceae bacterium oral taxon 096 (p <0.05), Lachnoanaerobaculum sp. S6-P3 (p <0.05), Neisseria perflava (p <0.05), Enterobacter sp. AR451 (p <0.05), Neisseria sp. 260 (p <0.05), Campylobacter showae (p <0.05), Stenotrophomonas maltophilia (p <0.05), Haemophilus sp. T13 (p <0.05), Treponema maltophilum (p <0.05), Halospirulina sp. EA11(2012) (p <0.05), bacterium MS4 (p <0.05), Neisseria flavescens (p <0.05), Leptotrichia sp. oral taxon 221 (p <0.05), Klebsiella pneumoniae (p <0.05) (species) - Increase in abundance in tongue coating in placebo group of Gemella sanguinis (p <0.05), Actinomyces sp. 'ARUP UnID 85' (p <0.05), Actinomycetaceae bacterium 'ARUP UnID 87' (p <0.05), Clostridiales bacterium oral taxon 085 (p <0.05), Streptococcaceae bacterium 'ARUP UnID 627' (p <0.05), Streptococcus sp. oral taxon C14 (p <0.05), Actinomyces sp. oral taxon 172 (p <0.05), Veillonella parvula (p <0.05), Leptotrichia sp. oral taxon 213 (p <0.05), Neisseria meningitides (p <0.05), Bacteroides plebeius (p <0.05) (species) - Decrease in abundance in tongue coating in placebo group of Leptotrichia wadei (p <0.05), Leptotrichia sp. oral taxon 223 (p <0.05), Stenotrophomonas maltophilia (p <0.05), Lachnoanaerobaculum orale (p <0.05) (species)

Some studies in model organisms also suggest that LF may have therapeutic utility in the field of neurodegenerative diseases. In mouse models of AD, nasal and oral application of LF led to reduced cognitive decline, lower concentration of Aβ and changes in protein expression of the brain, suggesting reduced inflammatory conditions and oxidative stress (Guo et al., 2017; Abdelhamid et al., 2020). In a nematode-model LF showed a reduction in paralysis as a result of the toxicity of Aβ, extended lifespan and upregulated genes involved in immune response, antioxidative defense, synaptic function and ubiquitin-mediated proteolysis (Martorell et al., 2017). However, further studies are required to better characterize the effect of LF in model organisms. The details of the studies are summarized in **Table 4**.

**Table 4:** Animal-studies on the therapeutic effects of LF on neurodegenerative diseases.

Author	Animal model	Intervention	Control	Duration	Measurements	Results
Guo et al., 2017	APPsw/PS1DE9 transgenic mice as AD model	Human LF, intranasal, 2mg/kg, or 6mg/kg Once per day	Saline vehicle	12 weeks	Morris water maze test, immunohistochemistry, immunofluorescence and western blot for several AD-related pathways, real-time PCR for cytokine expression and ROS formation assay of brain tissue	- Increased bodyweight in LF group (p <0.05) - Improved spatial learning impairment in LF group shown as decrease in escape latency (p <0.05) and increased passing time (p <0.01) - Reduced immunoreactivity of astrocyte marker GFAP in LF group (p <0.05) - In western blot increased level of synaptophysin (p <0.01), APP cleavage enzymes ADAM10 (p <0.05) and PS1 (p <0.01), sAPPα (p <0.05) and concomitant membrane-bound C-terminal fragment C83 (p <0.01), HIF1α (p <0.05), VEGF (p <0.01), phosphorylated ERK1/2 (p <0.01), phosphorylated CREB (p <0.01), LF (p <0.01) and LF receptor (p <0.01) and dereased level of APP (p <0.05 for low dose LF, p <0.01 for high dose LF) and TNFα (p <0.05) in LF group - Increase in level of SOD1 (p <0.01) and concomitant decrease on ROS level (p <0.05) in LF group - Decrease in mRNA expression of TNFα (p <0.05) and IL-6 (p <0.01) in LF group
Martorell et al., 2017	Transgenic Aβ1-42 Caenorhabditis elegans CL4176 as AD model	Liposomal, bovine LF added to the surface of growth medium plates	Wild type Caenorhabditis elegans as negative controls Gingko biloba extract EGb 761 (for paralysis assay) and Vitamin C (for oxidative stress assay) as positive controls	–	Life span, paralysis assay for Aβ toxicity in nematode muscle tissue, oxidative stress assay, microarray analysis of gene expression	- Delayed paralysis in LF group (p <0.0001) better than positive control - Increased survival under oxidative stress in LF group (p <0.001) better than positive control - Increased lifespan in LF group (p = 0.0006) - In microarray analysis upregulation of genes related to immune system stimulation, oxidative stress response, protein homeostasis, cellular adhesion and neurogenesis in LF group (p <0.05)
Abdelhamid et al., 2020	J20 PDGF-APPSw, Ind transgenic mice as AD model	Diet with 2% bovine LF, or 0.5% pepsin-hydrolyzed bovine LF	LF-free control diet	12 weeks	Novel object recognition test, Aβ ELISA and western blot for several AD-related pathways of brain tissue	- Reduced memory impairment in both LF groups shown as longer exploration time (p <0.05) and higher preference index (p <0.05) - In ELISA decreased level of soluble and insoluble Aβ in both LF groups in Cortex and hippocampus (p <0.05) - In western blot decreased level of BACE1 (p <0.05, p <0.05), sAβPPβ (p <0.05, p <0.001) and CTFβ (p <0.05, p <0.001) and increased level of sAβPPα (p <0.05, p <0.05), extracellular APOE (p <0.01, p<0.05) and ABCA1 (p <0.05, p <0.05) in both LF groups
Liu et al., 2020	MPTP treated mice as Parkinson model	Holo- or apo-LF, intragastric, in varying dosage, prior to intraperitoneal injection of MPTP	Untreated mice as negative control MPTP without LF as positive control	7 days	Pole test, iron staining, immunohistochemistry of LF receptor positive-stained neurons, immunofluorescence staining of dopaminergic neurons, HPLC of dopamine and western blot for Parkinson related pathways of brain tissue, ELISA of serum iron, ferritin and total iron binding capacity	- Decreased MPTP-induced prolonged climbing pole time in LF groups (p <0.05) - Increased LF expression in groups treated with MPTP (p <0.01) - Increased LF receptor expression in groups treated with LF, MPTP, or both (p <0.01) - Decreased MPTP-induced spleen weight loss (p <0.01), loss of dopaminergic neurons (p <0.001), depletion of dopamine (p <0.05), increase of DMT1 (p <0.05), decrease of Fpn1 (p <0.05), decrease of Bcl-2 (p <0.05), decrease in Bcl-2/Bax-ratio (p <0.01), increase of cleaved caspase 3 (p <0.01), decrease of Cu/Zn SOD (p <0.05) and iron disturbances in peripheral blood (p <0.05) in LF groups - Decreased iron-positive-stained cells in LF groups (p <0.001)
Zhou et al., 2021	APP/PS1 transgenic mice as AD model Young and middle aged mice	Diet with 0.8% bovine LF	LF-free control diet	16 weeks	Morris water maze test, glucose and insulin tolerance test, western blot for several AD-related pathways of brain tissue, 16S rDNA sequencing of cecal content	- In western blot increased level of synptophysin in middle aged LF group (p <0.05) - No significant difference in diversity in young and middle aged LF and control groups (Simpson-/Shannon-Index) and richness in middle aged LF and control groups (Chao 1, ACE) - Higher richness in young LF group compared to control group (Chao 1 (p <0.05), ACE (p <0.05)) - Higher abundance in young LF group compared to control of Oscilibacter (p <0.05) (genus) - Lower abundance in young LF group compared to control of Bacteroides (p <0.05), Alistipes (p <0.05) (genus) - Lower abundance in middle aged LF group compared to control of Proteobacteria (p <0.05) (phylum), Oscillospira (p <0.05), Coprococcus (p <0.05), Ruminococcus (p <0.05) (genus) Linear discriminant analysis of effect size (LEfSe): - Enriched in young LF group compared to control group (LDA score threshold 2): Oscillibacter, Anaerotruncus, EF096579_g, EU454405_g, Mollicutes_RF39, EU474361_g, EU774448_g, EF096976_g (genus) - Enriched in young control group compared to young LF group (LDA score threshold 2): Gammaproteobacteria, Bacilli (class), Lactobacillales, Enterobacteriales, Bacillales, Sphingomonadales, Pseudomonadales, Pasteurellales (order), Lachnospiraceae_UCG_004, Pasteurellaceae, Planococcaceae, Lactobacillaceae, Moraxellaceae, Sphingomonadaceae, Acidaminococcaceae, Enterobacteriaceae, Bacteroidaceae (family), Veillonella, Megasphaera, Pelomonas, Enhydrobacter, Acinetobacter, Haemophilus, Coprococcus_2, Aquabacterium, Alloprevotella, Lysinibacillus, Lactobacillus, Streptococcus, Phascolarctobacterium, Bacteroides, Escherichia_Shigella, EF602805_g, DQ809097_g, HM480175_g, EU506739_g, FJ504028_g, EF604701_g (genus) - Enriched in middle aged LF group compared to control group (LDA score threshold 2): Bacteroidetes (phylum), Bacteroidia (class), Methylobacterium (genus) - Enriched in midlle aged control group compared to middle aged LF group (LDA score threshold 2): Roseburia, Oscillospira (genus)

Finally, one clinical trial tried to investigate the therapeutic effect of LF in AD patients. Mohamed et al. designed a randomized, controlled trials with 50 patients (28 men, 22 women) with a clinical diagnosis of AD and a concordant MRT brain scan. They received 250mg of bovine LF per day or only the standard therapy. After three months, cases showed better results in the MMSE and AD assessment scale-cognitive subscale 11-item (ADAS-COG-11), higher concentrations of ACh and 5-HT, lower serum levels of markers of oxidative stress and inflammation, Aβ-42 and p-tau, a higher level of glutathione and changes in gene expression of peripheral blood lymphocytes (Mohamed et al., 2019).

## Conclusion

In this review we summarized and discussed the evidence for a connection between the oral microbiota and the pathogenesis of AD. AD is characterized by cognitive decline and the aggregation of amyloid and tau. Due to a demographic shift associated with ageing populations in developed and developing countries, AD represents one of the most pressing health issues of the world.

The ‘amyloid hypothesis’ of AD is supported by a recent clinical trial demonstrating efficacy of an antibody against soluble amyloid-beta (van Dyck et al., 2022). The underlying causes of amyloid agglomeration and inflammation in the brain, however, remain to be elucidated.

Various lines of evidence support the potential relevance of oral microbiota in this context. These include (i) evidence for increased rates of oral pathologies, in which the oral microbiota play an important role, in AD patients; (ii) evidence for an association between AD and specific oral pathogens, in particular PG; (iii) evidence for oral microbiome community shifts in AD patients. It is important to note, however, the potentially confounding role of reverse causality, such as changed patterns of personal oral hygiene due to the onset of dementia or pre-dementia, and the limitations of cross-sectional study designs and 16S rDNA sequencing approaches.

**Figure 1:**
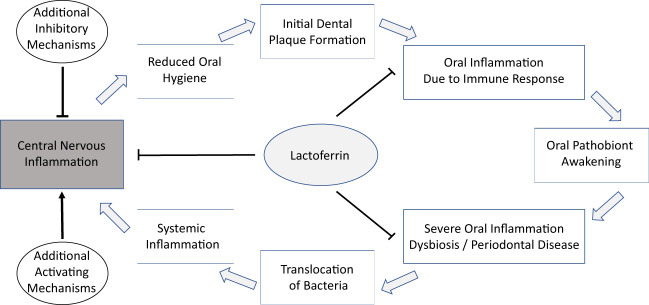
A hypothetical connection of the oral microbiome and AD might be a multi-step process with LF as a possible component in different stages and anatomical locations.

On a functional level, a link between the oral microbiota and AD could be mediated by (i) the presence of viable oral-associated bacteria in the brain; or (ii) the translocation, possibly mediated by outer-membrane vesicles, of inflammation-inducing or beta-amyloid-aggregation-inducing bacterial material, such as gingipain or LPS (Nie et al., 2019), to the brain. Both types of functional mechanisms are similar in that (i) they are compatible with the reported increased detection of microbial DNA and proteins, including DNA and proteins of the oral dybiosis-associated keystone pathogen *P. gingivalis*, in the brains of AD patients; and in that (ii) they both involve an important role for oral dysbiosis, which is associated with injuries of mucosal connective tissues, likely increasing the probability of translocation of both bacterial materials and intact bacteria from the mouth. Furthermore, the protein Lactoferrin, which occurs orally as well as in the brain, and which exhibits direct antimicrobial activity and influences iron homeostasis, may play an important link between the oral microbiota and inflammatory conditions of the host. This hypothetical connection is illustrated in **Figure 1**. It is important to note that all of these discussed mechanisms are fully compatible with the ‘amyloid hypothesis’.

Oral dysbiosis and the occurrence of oral pathologies are increasingly understood as polymicrobial conditions, which involve a complex interplay between key pathogens such as PG and other, individually less pathogenic, bacteria. While studies of the oral microbiome structure and of the occurrence of PD in AD patients strongly support the hypothesis that AD is associated with both shifts in oral microbiome structure and oral pathologies, no clear picture of an ‘oral microbiome signature’ of AD has emerged yet.

It is important to note that, as it stands, the potential connections discussed in this review between AD and the oral microbiome and PG, as well as between AD and LF, are still hypothetical in nature. As we have shown, clinical studies are inconsistent with respect to specific microbiome features reported to be associated with AD; apart from the finding that the oral microbiomes of AD patients seem to differ from the oral microbiomes of controls, no clear microbiome signature associated with AD has been identified so far. Important limitations of most studies include a cross-sectional design and reliance on 16S rDNA sequencing, which is prone to false-positives; reported results have an observational character at best and should be interpreted with caution.

With these limitations in mind, we recommend that future studies aiming to elucidate the association between AD and the oral microbiota should include longitudinal study designs and employ multiple and complementary approaches to the characterization of oral microbiomes, including shotgun metagenomics. In addition to providing more accurate and robust estimates of community composition, metagenomics approaches can also yield insights into the functional capabilities of microbial communities, which may also play a role in the development of AD. We also recommend that future studies cover different niches of the oral cavity as well as the cerebral fluid, employing consistent methodologies for the detection and quantification of microbial signatures. Key questions include whether the pathogenesis of AD involves the presence of intact oral bacteria in the brain, whether it is possible to identify AD-promoting oral microbiome signatures and, indeed, whether therapeutic interventions targeting the oral microbiome may influence AD risk or prognosis.

## Author contributions

CW, AD, and PF, text. CW, figures. All authors contributed to the article and approved the submitted version.
